# Supplementary material for: Tri‐Culture System Reveals an Activation Cascade From Microglia Through Astrocytes to Neurons During Neuroinflammation
Source: J Neurochem. 2026 Mar 20;170(3):e70412. doi: 10.1111/jnc.70412 (PMC13003425; doi:10.1111/jnc.70412)
Supplement: Supplementary file 1 — Data S1: jnc70412‐sup‐0001‐Supinfo1.pdf. [file JNC-170-0-s002.pdf]

Supplemental Information

Tri-culture system reveals an activation cascade from microglia through astrocytes to neurons during neuroinflammation

Hayato Kobayashi, Hiroshi Kato, Mitsuho Taniguchi, Setsu Endoh-Yamagami*

Bio Science & Engineering Laboratories, FUJIFILM Corporation, Kanagawa, Japan

Contact
*Correspondence: setsu.endo@fujifilm.com

Supplemental Figure 1

(A)

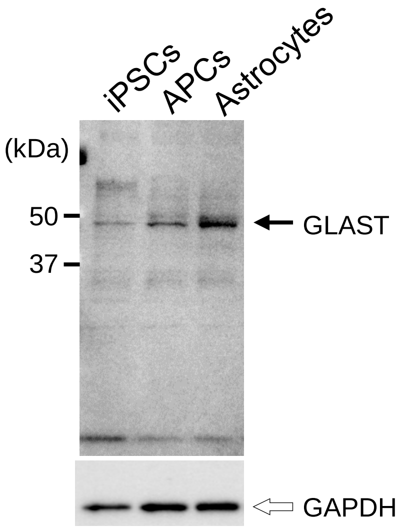

(B)

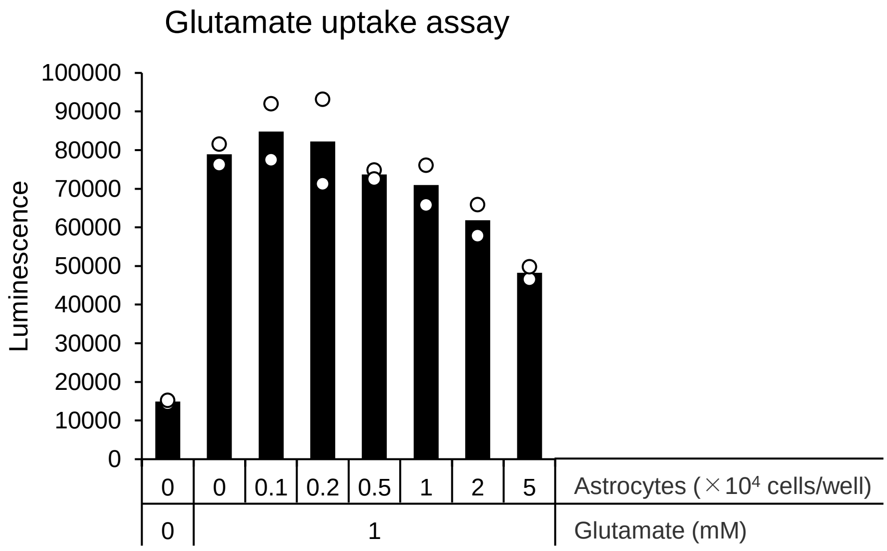

Supplemental Figure 1 GLAST expression and glutamate uptake ability in APCs and astrocytes. (A) Western blot analysis of GLAST expression in APCs and astrocytes (black arrow). The white arrow indicates GAPDH used as a loading control.

(B) The concentration of glutamate in the culture medium was measured 48 hours after glutamate supplementation. Increasing cell numbers resulted in a reduction in medium glutamate levels (data are presented the average of data from 2 wells).

Supplemental Table 1
Statistical summary for qRT-PCR data in Fig. 1B.

(A)

Gene	Comparison	t-value	df	p-value
S100B	iPSCs vs Astrocytes	44.60	4	<0.0001
KCNJ10	iPSCs vs Astrocytes	42.66	4	<0.0001
AQP4	iPSCs vs Astrocytes	59.43	4	<0.0001
SLC1A2	iPSCs vs Astrocytes	14.95	4	0.0001
SLC1A3	iPSCs vs Astrocytes	12.18	4	0.0003
GFAP	iPSCs vs Astrocytes	22.40	4	<0.0001

Note: Statistical analysis was performed using a two-tailed unpaired t-test. Values indicate t values, degrees of freedom (df) and p values. $p < 0.05$ was considered statistically significant.

Supplemental Table 2
Statistical summary for cytokine quantification assays in Fig. 1D.

(A)

Protein	Source	F	df	p-value
TNF-α	Factor A (culture type)	47.10	1, 7	0.0002
	Factor B (condition)	44.45	1, 7	0.0003
	Interaction	44.66	1, 7	0.0003
IL-1β	Factor A (culture type)	77.03	1, 7	<0.0001
	Factor B (condition)	42.84	1, 7	0.0003
	Interaction	57.30	1, 7	0.0001

(B)

Protein	Comparison	p-value
TNF-α	Monoculture_Vehicle vs. Tri-culture_Vehicle	>0.9999
	Monoculture_Vehicle vs. Monoculture_LPS	>0.9999
	Monoculture_Vehicle vs. Tri-culture_LPS	0.0001
	Tri-culture_Vehicle vs. Monoculture_LPS	>0.9999
	Tri-culture_Vehicle vs. Tri-culture_LPS	0.0002
	Monoculture_LPS vs. Tri-culture_LPS	0.0001
IL-1β	Monoculture_Vehicle vs. Tri-culture_Vehicle	>0.9999
	Monoculture_Vehicle vs. Monoculture_LPS	>0.9999
	Monoculture_Vehicle vs. Tri-culture_LPS	<0.0001
	Tri-culture_Vehicle vs. Monoculture_LPS	>0.9999
	Tri-culture_Vehicle vs. Tri-culture_LPS	<0.0001
	Monoculture_LPS vs. Tri-culture_LPS	<0.0001

Note: Statistical significance was determined by 2-way ANOVA followed by the Bonferroni's post-hoc test. Values indicate F values (F), degrees of freedom (df) and p values. $p < 0.05$ was considered statistically significant..

Supplemental Table 3
Statistical summary for Ca^{2+} imaging assays in Fig. 6A.

(A)

Culture type	Source	F	df	p
Co-culture	Factor A (experiments)	0.2249	2, 40	0.7996
	Factor B (condition)	29.09	2, 40	<0.0001
Tri-culture	Factor A (experiments)	0.3952	2, 40	0.6761
	Factor B (condition)	9.780	2, 40	0.0003

(B)

Culture type	Comparison	p-value
Co-culture	Vehicle vs LPS	0.9449
	Vehicle vs TNF-α	<0.0001
Tri-culture	Vehicle vs LPS	0.0207
	Vehicle vs TNF-α	0.0002

Note: Statistical significance was determined by 2-way ANOVA followed by the Dunnett's post hoc test. Values indicate F values (F), degrees of freedom (df) and p values.. $p < 0.05$ was considered statistically significant.

Supplementary Materials and Methods

Western blotting

Cell lysates for Western blotting were prepared by lysing cells with RIPA Lysis and Extraction Buffer (Thermo Fisher Scientific, cat. no. 89901), containing protease and phosphatase inhibitors (Thermo Fisher Scientific, cat. no. 1861280). Samples were then denatured for 5 minutes at 95° C with LDS sample buffer (Thermo Fisher Scientific, cat. no. NP0007), containing DTT (FUJIFILM Wako Pure Chemical, cat. no. 044-33871) at a final concentration of 100 mM. Protein samples were loaded onto 15% polyacrylamide gels (FUJIFILM Wako Pure Chemical, cat. no. 190-15001) and transferred onto 0.2 µm pore-size PVDF membranes (Bio-Rad, cat. no. 1704156) using the TurboBlot system (Bio-Rad). Subsequently, membranes were blocked with PVDF Blocking Reagent (TOYOBO, cat. no. NYPBR01) for 2 hours at room temperature. The membranes were incubated overnight at 4° C with primary antibodies against GLAST (Sigma, cat. no. MABN794) or GAPDH (Cell Signaling Technology, cat. no. 97166) in Can Get Signal Immunoreaction Enhancer Solution 1 (TOYOBO, cat. no. NKB-101), followed by incubation with secondary anti-mouse IRDye 800CW (abcam, cat. no. AB216772) antibodies in Can Get Signal Immunoreaction Enhancer Solution 2 (TOYOBO, cat. no. NKB-101) for 2 hours at room temperature.

Evaluation of glutamate uptake ability

The concentration of glutamate in the culture medium was determined using the Glutamate-Glo Assay Kit (Promega, cat. no. J7021), following the manufacturer's instructions. Astrocytes were cultured at defined densities ($0.1\text{-}5 \times 10^4$ cells/well) in a 96-well plate, and the medium was supplemented with 1 mM glutamate. After 48 hours of incubation, the culture supernatants were collected, and glutamate concentrations were quantified .
